# Changes in duodenal tissue-associated microbiota following hookworm infection and consecutive gluten challenges in humans with coeliac disease

**DOI:** 10.1038/srep36797

**Published:** 2016-11-09

**Authors:** Paul Giacomin, Martha Zakrzewski, Timothy P. Jenkins, Xiaopei Su, Rafid Al-Hallaf, John Croese, Stefan de Vries, Andrew Grant, Makedonka Mitreva, Alex Loukas, Lutz Krause, Cinzia Cantacessi

**Affiliations:** 1 Centre for Biodiscovery and Molecular Development of Therapeutics, Australian Institute of Tropical Health and Medicine, James Cook University, Cairns, Australia; 2 Bioinformatics Laboratory, QIMR Berghofer Medical Research Institute, Brisbane, Australia; 3 Department of Veterinary Medicine, University of Cambridge, Cambridge, United Kingdom; 4 Prince Charles Hospital, Brisbane, Australia; 5 McDonnell Genome Institute, Washington University School of Medicine, St. Louis, United States of America; 6 Department of Medicine, Washington University School of Medicine, St. Louis, United States of America; 7 The University of Queensland Diamantina Institute, Translational Research Institute, Woolloongabba 4102, Brisbane, QLD, Australia

## Abstract

A reduced diversity of the gastrointestinal commensal microbiota is associated with the development of several inflammatory diseases. Recent reports in humans and animal models have demonstrated the beneficial therapeutic effects of infections by parasitic worms (helminths) in some inflammatory disorders, such as inflammatory bowel disease (IBD) and coeliac disease (CeD). Interestingly, these studies have described how helminths may alter the intestinal microbiota, potentially representing a mechanism by which they regulate inflammation. However, for practical reasons, these reports have primarily analysed the faecal microbiota. In the present investigation, we have assessed, for the first time, the changes in the microbiota at the site of infection by a parasitic helminth (hookworm) and gluten-dependent inflammation in humans with CeD using biopsy tissue from the duodenum. Hookworm infection and gluten exposure were associated with an increased abundance of species within the Bacteroides phylum, as well as increases in the richness and diversity of the tissue-resident microbiota within the intestine, results that are consistent with previous reports using other helminth species in humans and animal models. Hence, this may represent a mechanism by which parasitic helminths may restore intestinal immune homeostasis and exert a therapeutic benefit in CeD, and potentially other inflammatory disorders.

A number of studies have reported the beneficial effects of experimental helminth infections on the pathology of a range of human autoimmune and allergic inflammatory disorders of the gastrointestinal tract, including inflammatory bowel disease (IBD) and coeliac disease (CeD)^1^,^2^,^3^,^4^,^5^,^6^. Indeed, according to the ‘Hygiene Hypothesis’ and the ‘Old Friends Theory’, exposure to pathogens (including parasitic helminths) during childhood is important for the development of regulatory immune mechanisms that, in turn, contribute to the prevention of diseases associated with inappropriate immune reactions against harmless stimuli^7^. As a consequence, controlled infections with selected parasitic helminths, such as whipworms and hookworms, have been proposed as an alternative therapeutic strategy (‘helminth therapy’) against some of these diseases^5^,^6^,^8^. For instance, experimental infections with the human hookworm *Necator americanus* have shown promise as a potential novel treatment for CeD^4^. Improved gluten tolerance following hookworm infection was associated with suppressed pro-inflammatory cytokine responses and increasing regulatory immune cell responses^4^. However, the biological and molecular mechanisms by which hookworms can suppress autoimmune diseases remain unclear and require further investigations.

One of these potential mechanisms is likely to rely on the production of immune-modulatory excretory/secretory products (ES) by hookworms, which include homologues of mammalian C-type lectins, galectins and cytokines^9^,^10^,^11^; however, it is likely that other biological and environmental factors are involved in these processes. In particular, given the primary role played by gastrointestinal dysbiosis in the pathogenesis of CeD^12^, it has been proposed that one of the mechanisms by which hookworms can support intestinal immune homeostasis in inflammatory disorders (such as CeD), is *via* the alteration of the composition of the gut microbiota and relative abundance of individual microbial species^8^,^13^,^14^,^15^,^16^,^17^. This hypothesis is based on the results of recent studies by us and others, in which experimental infections with gastrointestinal helminths were accompanied by detectable changes in commensal bacterial composition of both human and animal hosts^13^,^14^,^17^,^18^,^19^,^20^, as well as of the metabolic profiles of bacterial communities which indirectly promote the development of host regulatory T-cell responses^21^. In our previous study, experimental hookworm infection of human volunteers with CeD and administration of progressively increasing doses of dietary gluten resulted in maintenance of the composition of the gut flora, as determined from faecal samples from these subjects^14^. However, we also observed a significant increase in microbial species richness over the course of the trial^14^, which is generally associated with a ‘healthier’ intestinal status^22^. These findings were based on analyses of bacterial community profiles from faecal samples of CeD subjects^14^. While the collection of such samples presents numerous practical advantages, the nature of the microbiota within the faeces may not accurately reflect the dynamic changes in bacterial communities that may occur in the duodenal tissue following hookworm infection or gluten challenge. Therefore, the aim of this study was to investigate alterations in mucosally-associated (duodenal) microbiota of CeD subjects prior to and following experimental infection with *N. americanus*, as well as following the administration of a low (10–50 mg/day) and high (350 mg/day) dose of dietary gluten.

## Results

### Comparison of mucosally-associated bacteria in active Marsh 3 grade CeD Control and Trial subjects over the course of the experiment

**Figure 1:**
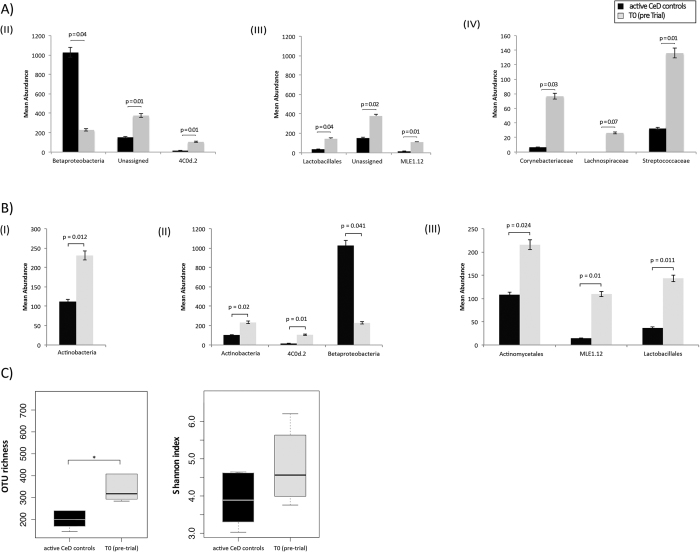
Differences between the mucosally-associated microbiota of Trial and Control subjects, and of Trial subjects over the course of the experiment. Differences in abundance of mucosally-associated bacteria (at the phylum- I, class – II, order - III and family – IV level) between Trial subjects prior to hookworm infection (T0) and of active coeliac disease Control subjects, based on LDA Effect Size (LEfSe) analysis (**A**) and paired t-test (**B**), with differences pre FDR indicated by the respective p-values. (**C**) Differences in overall taxonomic species richness (left panel) and diversity (right panel) between the mucosally-associated microbiota of Trial subjects prior to hookworm infection (T0) and that of Control subjects with active coeliac disease. Significant differences are indicated with asterisks (*p < 0.05).

All six Trial subjects included in the present study had been on a strict gluten free diet (GFD) for >5 years and had recorded sub-clinical Marsh scores prior to the beginning of the study; all were successfully infected with *N. americanus,* with parasite eggs detectable in faeces from 8–52 weeks post-infection^4^. Duodenal biopsy samples were collected from these subjects prior to the infection (T0), as well as following the administration of 10–50 mg/day gluten from weeks 12–24 (T24) and twice-weekly 1 g/day gluten from weeks 24 to 36 (approximately 350 mg gluten/day) (T36)^4^. Biopsy samples from six Control subjects from the same metropolitan area with active Marsh 3+ grade CeD were included for comparative purposes (Supplementary Table S1). A total of 19,562,372 paired-end reads were generated from the 24 samples analyzed (per sample mean 815,099 ± 236,697) (not shown). After primer trimming, joining of paired-end reads and filtering of low-quality sequences, a total of 3,861,592 high-quality sequences were subjected to further bioinformatics analyses. Of these, 2,815,504 reads (~73%) were assigned to operational taxonomic units (OTUs) belonging to the families Bradyrhizobiaceae and Burkholderiaceae, respectively (not shown). Since, in accordance with previous reports^23^,^24^, these bacteria were identified as contaminants of ultrapure water systems and/or DNA extraction kits, reads assigned to these OTUs were computationally subtracted from the dataset. Despite the high proportion of contaminant sequences in our datasets, rarefaction analysis demonstrated that the sequence data well covered the microbial diversity of the samples. Indeed, the rarefaction curves generated following *in silico* subtraction of these sequences indicated that the vast majority of mucosally-associated bacterial communities were represented in the remaining sequence data, thus allowing us to undertake further analyses. The remaining 1,046,088 sequences were assigned to 5,338 OTUs and 6 bacterial phyla, respectively (Supplementary Dataset 1). The composition of the mucosally-associated microbiota of the Trial subjects over the course of the study, and of Control subjects is shown in Supplementary Figure S1. The phyla Proteobacteria, Bacteroidetes and Firmicutes were predominant in all samples analysed, the latter two also being identified as predominant in our previous investigation of the faecal microbiota of the same study subjects^4^,^13^,^14^. Significant differences in abundance of individual taxa at the class, order and family level were observed between Trial subjects at baseline T0 (i.e. prior to hookworm infection and exposure to escalating doses of gluten) and Control subjects (subjects with active CeD) by LDA Effect Size (LEfSe) analysis (FDR < 0.05) (Fig. 1A). Also, analysis by paired t-test identified differences in abundance of individual taxa at the phylum, class and order level between Control subjects and Trial subjects at T0, albeit these differences were not significant following p-value correction for multiple testing (FDR > 0.05). In particular, Actinobacteria (phylum), Actinobacteria, unclassified 4C0d.2 and Betaproteobacteria (class), Actinomycetales, unclassified MLE1.12 and Lactobacillales (order), showed a trend towards increased abundance in Trial subjects compared with Control subjects by paired t-test (Fig. 1B). OTU richness was significantly higher in Trial subjects at T0 when compared to Controls (p = 0.026, t-test) (Fig. 1C).

### Composition of the mucosally-associated microbiota of Trial subjects over the course of the experiment

**Figure 2:**
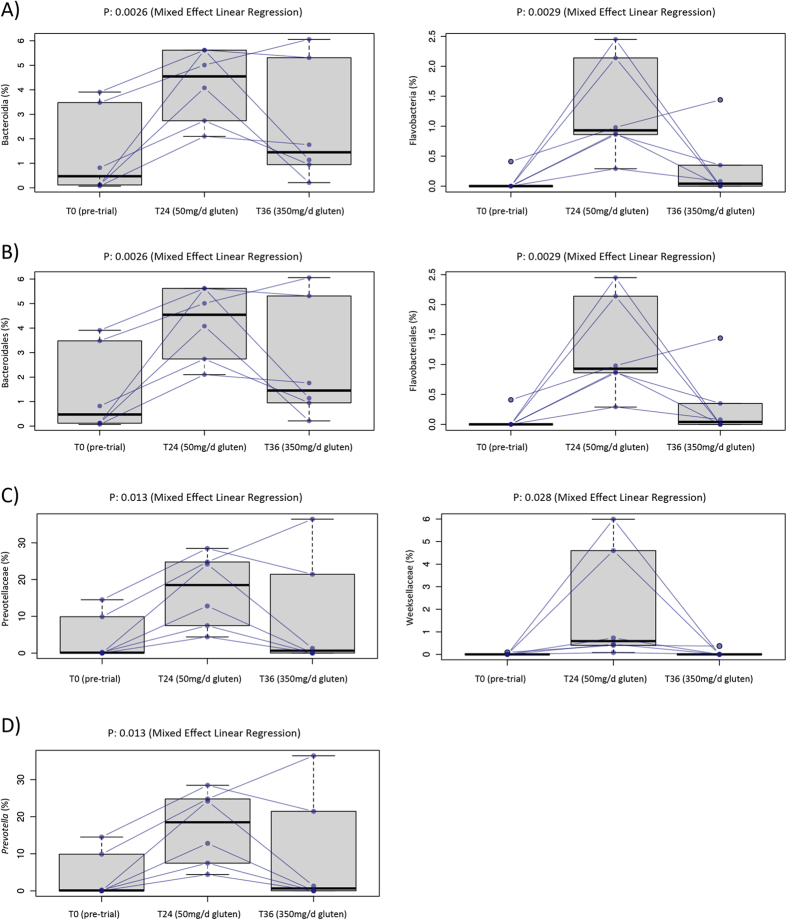
Differences between the mucosally-associated microbiota of Trial subjects over the course of the experiment. Differences in abundance of mucosally-associated bacteria, at the class (**A**), order (**B**), family (**C**) and genus (**D**) level, between the mucosally-associated microbiota of Trial subjects prior to hookworm infection (T0) and post-gluten challenge (T24 and T36).

Differences in the relative abundance of two classes, two orders, two families, one genus and 12 OTUs were observed in Trial subjects over the course of the experiment, using the mixed effect linear regression method (Fig. 2 and Supplementary Table S2); in particular, Bacteroidia and Flavobacteriia (class) and Bacteroidales and Flavobacteriales (order) displayed a trend towards increased abundance in Trial subjects at T24 compared with T0 (FDR < 0.05) (Fig. 2).

### Hookworm infection and gluten challenge are associated with a trend towards increased bacterial richness and diversity

**Figure 3:**
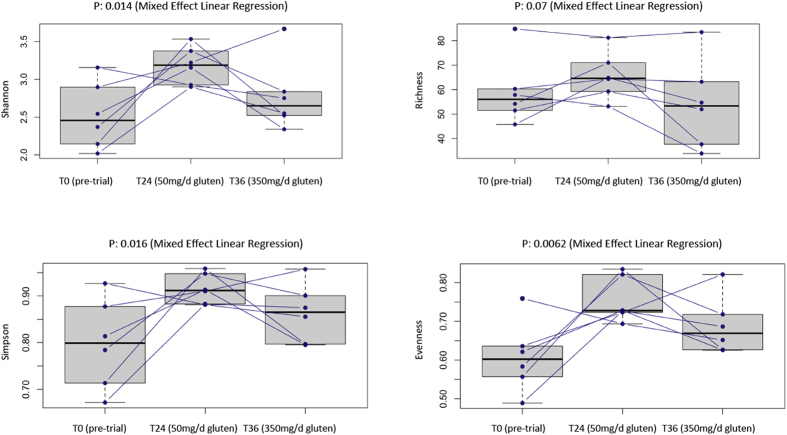
Trends towards increased bacterial richness and diversity are observed in Trial subjects post-hookworm infection and gluten challenge. Differences in overall taxonomic species richness and diversity between the mucosally-associated microbiota of Trial subjects prior to hookworm infection (T0) and post-gluten challenge (T24 and T36).

We next assessed longitudinal changes in microbial diversity in Trial subjects both pre-trial, and following hookworm infection and gluten challenge. Using linear mixed effects regression, we observed significant changes in microbial diversity (Shannon and Simpson index) and evenness over the course of the trial (Fig. 3). We also observed changes in OTU richness, which almost reached statistical significance (p = 0.07, Fig. 3). At week 24 (post-hookworm infection and gluten microchallenge) we observed a significant increase in OTU diversity (Shannon index p = 0.019, Simpson index p = 0.024; paired t-test) compared to T0 samples. At week 36 (post-350 mg/d gluten challenge), OTU diversity returned to baseline T0 levels (Fig. 3, paired t-test T0 *vs* T24, Shannon and Simpson index p > 0.5).

### Cluster analysis reveals time-point grouping of samples from Trial subjects

**Figure 4:**
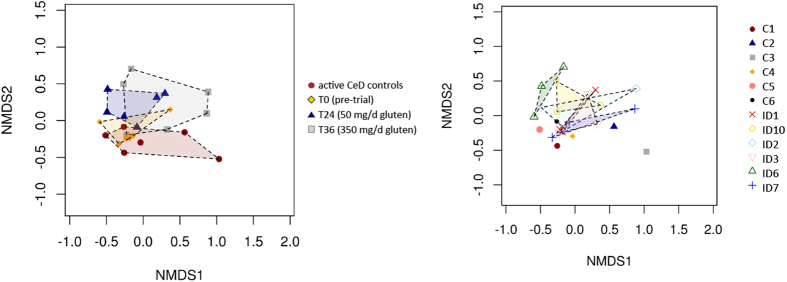
Unsupervised NMDS analysis. Unsupervised NMDS analysis of the composition of the mucosally-associated microbiota of Trial subjects prior to hookworm infection (T0) and following exposure to escalating doses of dietary gluten (T24 and T36, respectively), as well as of Control subjects with active coeliac disease, ordered by time point (left panel), and subject ID (right panel), respectively.

**Figure 5:**
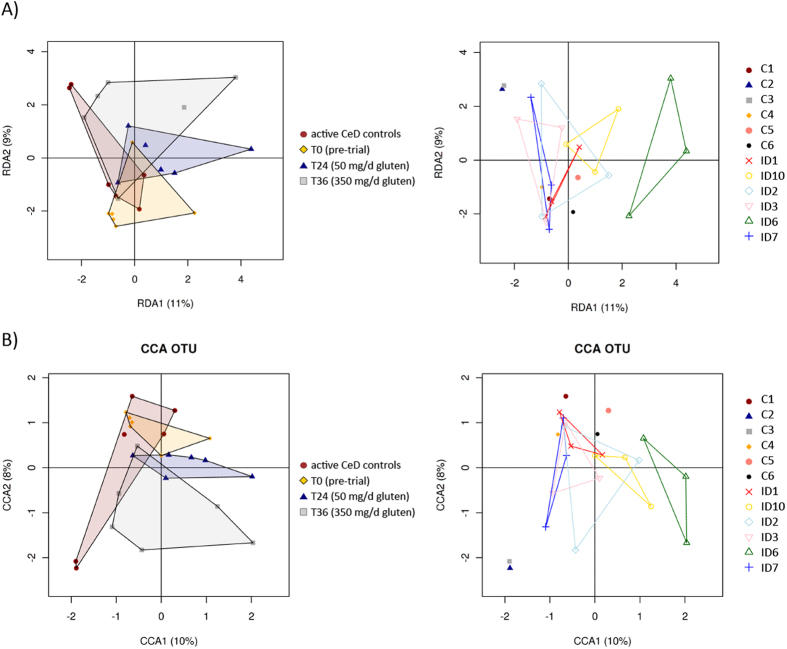
Supervised RDA and CCA analyses reveal clustering according to time points. Supervised RDA (**A**) and CCA (**B**) depicting the composition of the mucosally-associated microbiota of Trial subjects prior to hookworm infection (T0) and following exposure to escalating doses of dietary gluten (T24 and T36, respectively), as well as of Control subjects with active coeliac disease, ordered by time point (left panels), and subject ID (right panels), respectively.

Mucosally-associated microbial communities were grouped by hierarchical clustering and ordinated by unsupervised non-metric multidimensional scaling (NMDS). The latter revealed that microbial samples from Control and Trial subjects formed separate clusters and samples from Trial subjects showed a tendency to cluster by time point (Fig. 4). Redundancy analysis (RDA), canonical correlation analysis (CCA) and Adonis confirmed this observation, as all three methods identified a significant association between community composition and sample time point (RDA, CCA, Adonis; p ≤ 0.045). RDA and CCA were able to clearly separate biopsy samples by time point (cf. Fig. 5). These results suggest a correlation between infection status and exposure to escalating doses of gluten and the composition of the gut microbiota.

## Discussion

In this study we assessed, for the first time, the effects of experimental hookworm infection and gluten ingestion on the nature of the duodenal tissue-associated microbiota of CeD subjects. The unique opportunity to assess changes in bacterial communities at the site of hookworm infection and CeD-associated inflammation is of crucial importance for dissecting the putative direct and/or indirect role/s of the gut microbiota in helminth-driven suppression of inflammation. We observed qualitative and quantitative changes in the composition of the tissue-resident microbiota from subjects with active, Marsh 3+ grade CeD compared to the Trial subjects who had well-managed CeD due to long-term adherence to a GFD. Interestingly, hookworm infection and gluten exposure in Trial subjects was associated with fluctuations in bacterial species richness and diversity, and trends towards increased abundance of species within the Bacteroides phylum, similar to previous reports analyzing the faeces of helminth-infected humans and primates^14^,^18^. Together, these data suggest that helminth infections and gluten exposure can significantly alter the composition of the tissue-resident and faecal microbiota, which has implications for the purported therapeutic efficacy of helminths in inflammatory disease.

Given that CeD diagnosis necessitates the collection of gut biopsy tissue, several studies have used this opportunity to characterize the mucosal microbiota in CeD patients, allowing for direct comparison of faecal and tissue microbiota in some instances. While some studies have shown consistency between the faecal and tissue microbiota in CeD patients^25^, others have shown substantial differences^26^,^27^, which would be expected given the distinct anatomical locations^28^,^29^. These findings clearly demonstrate the utility of analyzing both the faecal and tissue microbiota where possible. In the present investigation, we extended on our previous published analyses of the faecal microbiota^13^,^14^; however, given the profound methodological differences between this and our previous works^13^,^14^, direct comparisons of the results obtained are unwarranted.

Here, we compared the composition of the mucosally-associated microbiota of Trial subjects, prior to hookworm infection and exposure to dietary gluten (T0), to that of Control subjects. The mucosally-associated microbiota of the former group showed a trend towards higher numbers of Actinobacteria (phylum level), Actinobacteria and the unclassified 4C0d.2 (class), and Actinomycetales, the unclassified MLE1.12 and Lactobacillales (order) (cf. Fig. 1). The phylum Actinobacteria is one of the most abundant phyla in the gut commensal flora of humans^30^, and is enriched in the upper intestine when compared to other intestinal sites^31^. The phylum includes pathogens, such as the genus *Mycobacterium*, responsible for a diverse range of diseases in humans and animals, and gastrointestinal commensals, such as bacteria within the family Bifidobacteriaceae, which are characterized by well known probiotic properties due to their ability to ferment oligosaccharides^32^, as well as to modulate the immune system of their human hosts^33^. Increased concentrations of bifidobacteria have been reported in the duodenum of children with non-active CeD (i.e. on a strict GFD since > 2 years) when compared with both children with active CeD and healthy controls; such observations led to the hypothesis that these bacterial populations may be partially restored following the introduction of a GFD^25^. However, in another study, the differences in concentration of duodenal Actinobacteria between CeD patients on a GFD with and without persistent symptoms were statistically insignificant^34^. Among others, methodological differences between these studies, i.e. bifidobacteria-targeted real-time PCR^25^ *versus* 16S rRNA gene pyrosequencing^34^, may have contributed to this discrepancy. The present study contained too few samples to allow us to draw definite conclusions about the effects of a GFD or the grade of active CeD on Actinobacteria populations in Trial and Control subjects. Similarly, lactobacilli appeared to be less prevalent in the active CeD Controls compared to our Trial subjects with diet-managed CeD. This group of bacteria is of particular interest in CeD research, as it includes known probiotics shown to exert a positive impact on a range of gastrointestinal inflammatory conditions, such as those caused by IBD and rotavirus infections^35^,^36^. Lactobacilli are decreased in the duodenal and faecal microbiota of CeD children (with active disease) compared with CeD children on a GFD, as well as with healthy controls^25^,^26^, which supports the hypothesis that the adherence to a strict GFD contributes to the restoration of a ‘healthy’ intestinal flora^25^.

We characterized the qualitative and quantitative fluctuations in the composition of mucosally-associated microbiota of Trial subjects experimentally infected with hookworms and exposed to progressively increasing doses of dietary gluten. Compared with biopsy samples collected from Trial subjects at T0 (i.e. prior to experimental infections), those collected following hookworm infection and gluten micro-challenge (T24) displayed higher relative abundances of the orders Bacteroidales and Flavobacteriales, of the Bacteroides phylum. Bacteria within this phylum were also increased in the faecal microbiota (assessed by 16S rRNA high-throughput sequencing) of macaques with idiopathic chronic diarrhea (ICD) after experimental infection with *Trichuris* whipworms, when compared with uninfected controls exhibiting clinical signs of disease^18^. However, in the same study, evaluation of absolute Bacteroidetes abundance by real-time PCR displayed a reduction in the populations of these bacteria in the faeces of macaques post-*Trichuris* treatment, which led the authors to hypothesize that the apparent increase observed by high-throughput sequencing of the 16S rRNA gene might have reflected an expansion of non-Bacteroidetes phyla in diseased subjects^18^. More recent studies in murine models and in human populations have supported a role for *Trichuris* in regulating the balance of Bacteroidetes within the gut, while simultaneously promoting Clostridiales colonization^17^. Nonetheless, in our study, a larger relative abundance of Bacteroidetes was detected in Trial subjects at both T0 and following the introduction of an inflammatory stimulus (i.e. gluten, T24). Based on our preliminary observations, as well as knowledge that these bacteria are more abundant in CeD subjects displaying no symptoms of disease compared with symptomatic controls^26^,^34^ we hypothesize that the relative expansion of populations of Bacteroidetes between T0 and T24 may be associated with helminth infections. Consistent with this, exposure to gluten challenges in trial subjects was not accompanied by an expansion of Betaproteobacteria that were abundant in the mucosally-associated microbiota of Control subjects with CeD. This finding leads us to hypothesize that hookworms may indeed contribute towards the maintenance of the gut homeostasis in presence of an inflammatory insult. However, these hypotheses require testing in larger, placebo controlled, clinical trials.

Fluctuations in overall bacterial diversity and richness were observed between biopsy samples from Control and Trial subjects at T0, and Trial subjects at T0, T24 and T36. In particular, richness was significantly higher in Trial subjects at T0 when compared to Controls, while samples from Trial subjects at T24 displayed a significant increase in OTU diversity compared to T0 samples. Diversity returned to baseline T0 levels at T36. The observed difference in bacterial richness between Control and Trial subjects at T0 is in overall agreement with the results of a number of previous studies on the composition of the gut microbiota of individuals suffering from a range of inflammatory intestinal disorders, including CeD and IBD^37^,^38^,^39^, and it generally reflects a ‘healthier’ intestinal homeostasis^40^. Similarly, the observed differences in bacterial diversity in Trial subjects at T24 compared with T0 is also in agreement with our previous observations on the faecal microbiota of the same subjects^14^, and supports our previous hypothesis that the ability of hookworms to act as a gluten-tolerising agents in CeD subjects^4^ may partly be linked to their direct and/or indirect capacity to promote the restoration of a healthy bacterial diversity and richness in the gastrointestinal tract^8^. In support of this hypothesis, a previous study by Broadhurst *et al.*^18^ also showed that the gut microbiota of *Trichuris*-infected macaques with ICD was characterized by a significantly higher bacterial diversity and richness when compared to uninfected controls, which the authors attributed to the symptomatic improvement observed in the former group^18^. Moreover, in a separate study investigating the composition of the faecal microbiota in humans naturally infected with gastrointestinal parasitic helminths, Lee *et al.*^41^ also detected a significantly higher bacterial richness in the microbiota of subjects from indigenous Malaysian communities with natural infections with *Trichuris* and/or hookworms and/or *Ascaris* sp. compared with uninfected subjects from the same geographical area^41^. It is also worth noting that, in the study by Broadhurst *et al.*^18^, the initial infection dose consisted of ~1000 *T. suis* ova and, while in the study by Lee *et al.*^41^ a precise estimation of the infection burdens affecting the subjects tested would be highly speculative, this is likely to be substantially higher than the hookworm infection dose that was inoculated into Trial subjects enrolled in our study (20 infective larvae overall). This observation further substantiates the role of hookworms as potent inducers of host responses^9^, which may also be reflected in their capacity to alter the gut microbiota of infected individuals. In a recent experiment, changes in metabolites produced by the gut microbiota of the same cohort of Trial subjects investigated in both this and our previous study^14^ were examined^21^. The results showed that the faecal samples of 4 out of 6 Trial subjects whose mucosally-associated microbiota was examined herein, were characterized by increased levels of short-chain fatty acids (SCFA), which were reflected by changes in the concentrations of acetate, propionate and butyrate) post-hookworm infection compared with T0 baseline levels^21^. These metabolites have been demonstrated to exert anti-inflammatory properties by promoting host regulatory T cell responses^21^,^42^,^43^,^44^,^45^. It is therefore plausible that factors linked to the increase in microbial diversity and richness, as well as in microbial anti-inflammatory metabolites contribute synergistically to the ability of hookworms to dampen inflammatory reactions and establish chronic infections in the human host. However, in our study, we also observed a significant decrease in bacterial diversity and richness at T36 (following gluten challenge) compared with T24 (following hookworm infection and gluten micro-challenge), albeit the latter difference was statistically insignificant. This finding may be associated with the onset of inflammatory responses that occurred in Trial subjects at T36, as a consequence of the substantial increase in the doses of dietary gluten to which they had been exposed (350 mg daily from T24 to T36 *vs* 10 to 50 mg daily up to T24)^4^. Nevertheless, in 5 out of 6 Trial subjects included in the present study, the duodenal villous height:crypth depth ratio (Vh:Cd), i.e. a measure of the mucosal inflammation and intestinal pathology^46^, was improved or unaltered following gluten challenge compared with biopsy samples collected post-gluten microchallenge (T24)^4^, possibly indicating that the composition of the gut microbiota reacts rapidly in response to dietary changes and/or inflammatory stimuli. Data on the levels of SCFA in faecal samples from Trial subjects following exposure to dietary gluten are unavailable^21^, therefore a correlation between such levels and the observed decrease in microbial diversity and richness at T36 could not be established.

Some limitations of our study, as for our previous investigations on the faecal microbiota of the same Trial subjects^4^,^13^,^14^, were (i) the small sample size, which may have prevented us from detecting significant differences in the composition of the mucosally-associated microbiota of hookworm-infected CeD subjects exposed to increasing doses of dietary gluten; (ii) the unavailability of duodenal biopsy samples collected following hookworm establishment but prior to gluten challenge, which affected our ability to clearly separate the effects of helminth infection and the introduction of an inflammatory stimulus on the composition of the mucosally-associated microbiota; and (iii) the absence of hookworm- and gluten-placebo cohorts of subjects. Therefore, while the observations from the present study are promising, larger placebo-controlled clinical trials are necessary to confirm or confute our hypotheses regarding the putative role(s) of the gut microbiota in such mechanisms.

## Methods

### Ethics statement

This study was approved and carried out in strict accordance and compliance with the National Statement on Ethical Conduct in Research Involving Humans guidelines of the National Health and Medical Research Council of Australia (NHMRC). The Prince Charles Hospital (Brisbane, Australia) and James Cook University Human Research Ethics Committees approved the study. Written informed consent was obtained from all subjects enrolled in the study. This study was registered as a clinical trial at ClinicalTrials.gov as NCT01661933^4^.

### Trial design

Six otherwise healthy volunteers with CeD (HLA-DQ2+ or HLA-DQ8+) on a strict GFD (>5 years) (=Trial subjects) were infected percutaneously with 20 infective third stage larvae of *N. americanus*^4^. Subjects then underwent exposure to escalating doses of dietary gluten (as spaghetti), with a 10–50 mg/day micro-challenge from weeks 12–24, followed by intermittent twice-weekly 1 g/day gluten challenge from weeks 24 to 36 (approximately 350 mg gluten/day)^4^. Prior to experimental infection (T0), as well as at 24 (T24) and 36 weeks (T36) post-infection, two individual biopsy samples were collected from a randomly selected region of the duodenum of each subject by an accredited gastroenterologist (J. Croese) supported by an anaesthetist in an accredited facility (Prince Charles Hospital, Brisbane, Australia) and stored at −80 °C in Trizol solution. In addition, individual duodenal biopsy samples from six hookworm-naïve volunteers with active CeD (diagnosed as Marsh grade 3 at the time of biopsy)^4^ (= Control subjects) were also included for comparative purposes. A list of subject IDs whose mucosally-associated microbiota were analysed in the present study is presented in Supplementary Table S1.

### DNA extraction and 16S Illumina sequencing

Genomic DNA was extracted directly from each sample, as well as from two negative controls, using the Trizol RNA/DNA extraction kit, according to manufacturers’ instructions. High-throughput sequencing of the V3-V4 hypervariable region of the bacterial 16S rRNA gene was performed on an Illumina MiSeq platform according to the manufacturers’ protocols with minor adjustments. Briefly, the V3-V4 region was PCR-amplified using universal primers^47^, that contained the Illumina adapter overhang nucleotide sequences, using the NEBNext hot start high-fidelity DNA polymerase (New England Biolabs) and the following thermocycling protocol: 2 min at 98 °C, 35 cycles of 15 s at 98 °C – 30 s at 63 °C – 30 s at 72 °C, and a final elongation of 5 min at 72 °C. Amplicons were purified using AMPure XP beads (Beckman Coulter) and the NEBNext hot start high-fidelity DNA polymerase was used for the index PCR with Nextera XT index primers (Illumina) according to the following thermocycling protocol: 30 s at 98 °C, 8 cycles of 10 s at 98 °C – 75 s at 65 °C, and 5 min at 65 °C. The indexed samples were purified using AMPure XP beads, quantified using the Qubit dsDNA broad range kit (Life Technologies), and equal quantities from each sample were pooled. The resulting pooled library was quantified using the NEBNext library quantification kit (New England Biolabs) and sequenced on an Illumina MiSeq platform using the v3 chemistry (301 bp paired-end reads). Raw sequence data have been deposited in the NCBI Sequence Read Archive database under accession number SRP078558.

### Bioinformatics analyses

Raw paired-end Illumina reads were trimmed for 16S rRNA gene primer sequences using Cutadapt ( https://cutadapt.readthedocs.org/en/stable/) and reads were joined using FLASH ( https://ccb.jhu.edu/software/FLASH/)^48^. Pre-processed sequence data were processed using the Quantitative Insights Into Microbial Ecology (QIIME) software suite^49^. Successfully joined sequences were quality filtered in QIIME using default settings. Then, sequences were clustered into OTUs on the basis of similarity to known bacterial sequences available in the Greengenes database (v13.8; http://greengenes.secondgenome.com/; 97% sequence similarity cut-off) using the UCLUST software; sequences that could not be matched to references in the Greengenes database were clustered *de novo* based on pair-wise sequence identity (97% sequence similarity cut-off). The first selected cluster seed was considered as the representative sequence of each OTU. Then, representative sequences were assigned to taxonomy using the UCLUST software. Singleton OTUs were removed prior to downstream analysis. Normalisation was carried out by generating a subsampled OTU table by random sampling (without replacement) of the input OTU table using an implementation of the Mersenne twister algorithm ( http://www.numpy.org/). Subsequently, OTU tables were rarefied to accommodate for different sampling depths. Samples characterised by fewer than the requested rarefaction depth (i.e. 10,666 sequences) were omitted from the output OTU table. Statistical analyses were executed in R version 3.1.2 ( http://www.r-project.org/); normality of variables was tested by Shapiro test and equality of variance by Levene test. The mean abundance of each taxon across different time points was analysed by Repeated Measured ANOVA for taxa with normal distribution and equal variance, and by the non-parametric Friedmann test for taxa for which these two assumptions were not fulfilled. Differences in abundance of individual taxa were assessed by paired t-test if the differences between the pairs were normally distributed and by Wilcoxon test for non-normally distributed differences between pairs. p-values were corrected for multiple testing by holm adjustment. Further statistical analyses were executed using the Calypso software (cgenome.net/calypso/); in particular, unsupervised hierarchical clustering, as well as NMDS analysis, were performed to obtain an overview of the distribution of samples according to sample origin and time points. ANOSIM was used to compare the overall composition of microbiota across subjects and time points. Supervised RDA (including subject and time point as explanatory variables), CCA, Adonis and Anosim were applied on the OTU table in the Calypso software with default parameters. Differences in the composition of the mucosally-associated microbiota between Control and Trial subjects (prior to hookworm infection and gluten challenge only, i.e. T0) were assessed using the LEfSe workflow^50^, by assigning ‘helminth infection status’ as comparison class. Changes in bacterial diversity and richness in Trial subjects over the course of the experiment (i.e. at T0, T24 and T36), as well as of longitudinal changes in the abundance of individual taxa, were evaluated using paired t-test and mixed effects linear regression (in order to account for correlations between repeated measures on the same subjects)^51^. Mixed effects linear regression models included taxa abundance (or diversity) as dependent variable, time point as fixed effect and individual as random effect.

## Additional Information

**How to cite this article**: Giacomin, P. *et al.* Changes in duodenal tissue-associated microbiota following hookworm infection and consecutive gluten challenges in humans with coeliac disease. *Sci. Rep.* **6**, 36797; doi: 10.1038/srep36797 (2016).

**Publisher’s note:** Springer Nature remains neutral with regard to jurisdictional claims in published maps and institutional affiliations.

## Supplementary Material

Supplementary Information

Supplementary File 1
