# Supplementary material for: Changes in duodenal tissue-associated microbiota following hookworm infection and consecutive gluten challenges in humans with coeliac disease
Source: Sci Rep. 2016 Nov 9;6:36797. doi: 10.1038/srep36797 (PMC5101533; doi:10.1038/srep36797)

Changes in duodenal tissue-associated microbiota following hookworm infection and consecutive gluten challenges in humans with coeliac disease

Paul Giacomin[1], Martha Zakrzewski[2], Timothy Jenkins[3], Xiaopei Su[3], Rafid Al-Hallaf[1], John Croese[4], Stefan de Vries[3], Andrew Grant[3], Makedonka Mitreva[5,6], Alex Loukas[1*], Lutz Krause[7*], Cinzia Cantacessi[1,3*]

Supplementary Table S1 | Trial subject identification (ID) numbers or control subject (C) numbers displaying age, sex, baseline clinical diagnosis (T0 Marsh Score). Corresponding subjects IDs as indicated by Zaiss *et al.*[20] are also indicated where applicable.

Subject	Age	Sex	Marsh score	Subject ID according to Zaiss *et al.*[20]
ID1	44	F	M0	Pt2
ID2	40	F	M0	Pt3
ID3	48	F	M0	Pt4
ID6	40	M	M0	Pt8
ID7	64	M	M0	Pt1
ID10	52	M	M2	Pt7
C1	57	F	M3b	n/a
C2	22	M	M3a	n/a
C3	48	F	M3a	n/a
C4	52	F	M3b	n/a
C5	51	M	M3a	n/a
C6	75	M	M3b	n/a

Supplementary Table S2 | Differentially abundant OTUs in the mucosally-associated microbiota of Trial subjects over the course of the study.

OTU	p-value	Adjusted p-value	FDR
Unassigned (kingdom)	0.0016	0.08	0.046
Ralstonia (genus)	0.0025	0.12	0.046
Chitinophagaceae (family)	0.0034	0.16	0.046
Chitinophagaceae (family)	0.041	0.19	0.046
Unassigned (kingdom)	0.0063	0.29	0.046
Unassigned (kingdom)	0.0066	0.3	0.046
Phyllobacterium (genus)	0.007	0.31	0.046
Unassigned (kingdom)	0.0074	0.32	0.046
Chitinophagaceae (family)	0.0092	0.39	0.05
Rhizobiales (order)	0.011	0.45	0.05
Rhizobiales (order)	0.011	0.45	0.05
Rhizobiales (order)	0.012	0.47	0.05

Supplementary Figure S1 | Microbial composition of duodenal biopsy samples, at the phylum (a) and genus (b) level, from Trial subjects (ID*) prior to and following hookworm infection (T0), as well as following administration of escalating doses of gluten (10-50 mg/day – T24; 350 mg/day – T36) as predicted in the analysis of the V1-V3 16S rRNA gene. Bubble sizes reveal the relative abundance (%) of phylotypes (based on 97% sequence identity) in each sample. The composition of the microbial communities detected in duodenal biopsy samples from Control subjects with active coeliac disease (C*) is also shown. OTUs listed were present in at least one sample at a relative abundance of ≥7%.

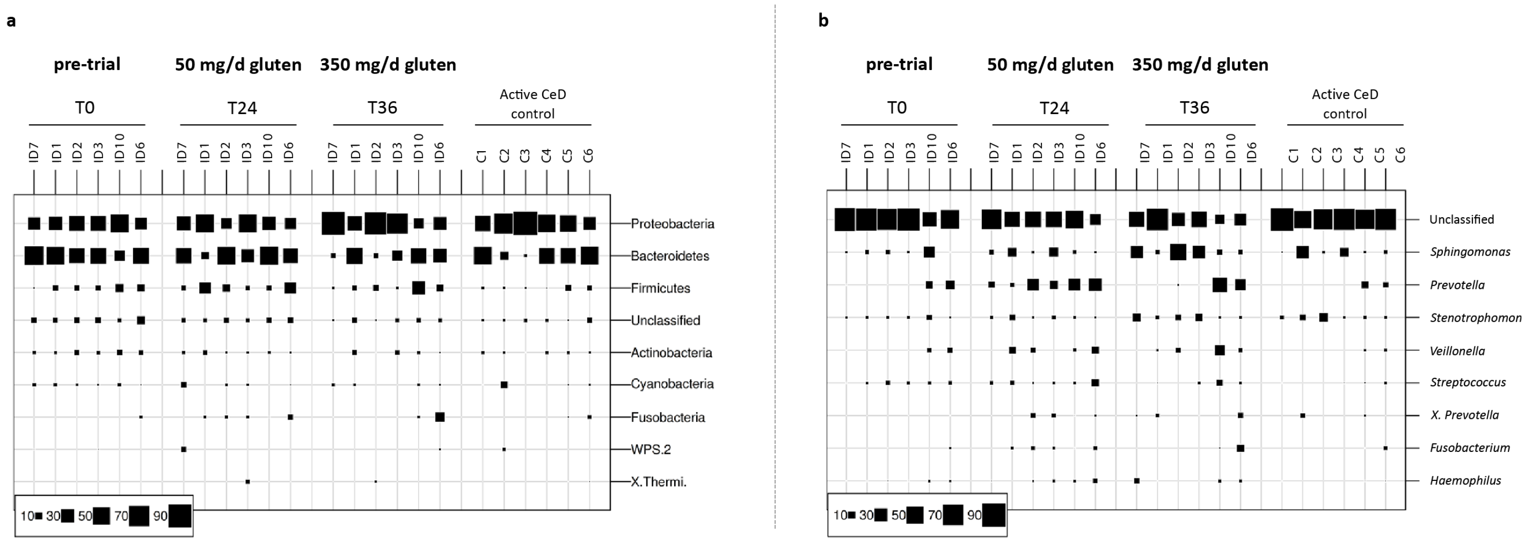

Supplementary Figure 2 | Hierarchical clustering heatmap of the composition of the mucosally-associated microbiota of Trial subjects prior to hookworm infection (T0) and following exposure to escalating doses of dietary gluten (T24 and T36, respectively), as well as of Control subjects with active coeliac disease. Dendrograms at the top of the heatmap indicate relationships between samples. Color intensity represents the relative abundance of sequences representing the corresponding bacterial family in each sample. Only OTUs with at least 0.5% relative abundance in at least one sample were included (174 OTUs in total). Data was scaled by row (OTU) and clustered using the R hclust.2() function (Euclidian distance and complete clustering method).

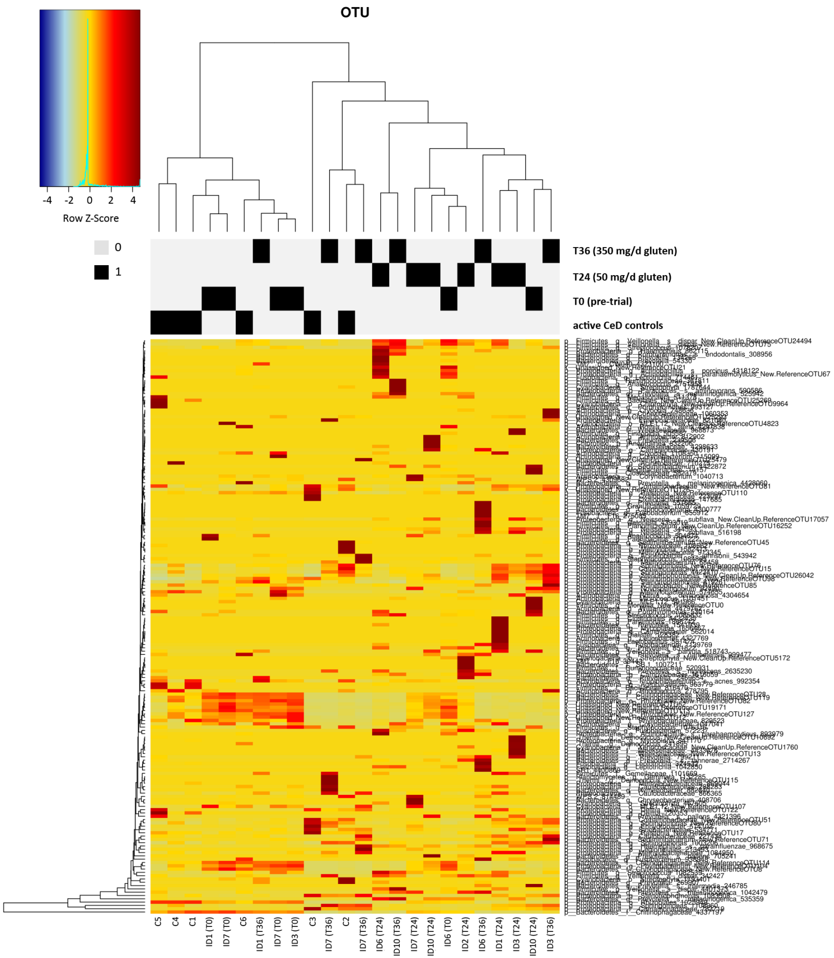

Supplement: Supplementary File 1 [file srep36797-s2.pdf]
